# How and why do win–win strategies work in engaging policy-makers to implement Health in All Policies? A multiple-case study of six state- and national-level governments

**DOI:** 10.1186/s12961-019-0509-z

**Published:** 2019-12-21

**Authors:** Lauri Kokkinen, Alix Freiler, Carles Muntaner, Ketan Shankardass

**Affiliations:** 1 Faculty of Social Sciences, Tampere University, Arvo Ylpön katu 34, 33014 Tampere, Finland; 2 Centre for Urban Health Solutions, Li Ka Shing Knowledge Institute, 209 Victoria Street, Toronto, Ontario M5B 1T8 Canada; 3 Dalla Lana School of Public Health, University of Toronto, 155 College Street, Toronto, Ontario M5T 3M7 Canada; 4 Bloomberg School of Nursing, University of Toronto, 155 College Street, Toronto, Ontario M5T 3M7 Canada; 5 Department of Health Sciences, Wilfrid Laurier University, 75 University Ave W, Waterloo, Ontario N2L 3C5 Canada

**Keywords:** Health in All Policies, multiple-case study, systems framework, realist methods, California, Ecuador, Finland, Norway, Scotland, Thailand

## Abstract

**Background:**

Much of the research about Health in All Policies (HiAP) implementation is descriptive, and there have been calls for more evaluative evidence to explain how and why successes and failures have occurred. In this cross-case study of six state- and national-level governments (California, Ecuador, Finland, Norway, Scotland and Thailand), we tested hypotheses about win–win strategies for engaging policy-makers in HiAP implementation drawing on components identified in our previous systems framework.

**Methods:**

We used two sources of data — key informant interviews and peer-reviewed and grey literature. Using a protocol, we created context–mechanism–outcome pattern configurations to articulate mechanisms that explain how win–win strategies work and fail in different contexts. We then applied our evidence for all cases to the systems framework. We assessed the quality of evidence within and across cases in terms of triangulation of sources and strength of evidence. We also strengthened hypothesis testing using replication logic.

**Results:**

We found robust evidence for two mechanisms about how and why win–win strategies build partnerships for HiAP implementation — the use of shared language and the value of multiple outcomes. Within our cases, the triangulation was strong, both hypotheses were supported by literal and contrast replications, and there was no support against them. For the third mechanism studied, using the public-health arguments win–win strategy, we only found evidence from Finland. Based on our systems framework, we expected that the most important system components to using win–win strategies are sectoral objectives, and we found empirical support for this prediction.

**Conclusions:**

We conclude that two mechanisms about how and why win–win strategies build partnerships for HiAP implementation — the use of shared language and the value of multiple outcomes — were found as relevant to the six settings. Both of these mechanisms trigger a process of developing synergies and releasing potentialities among different government sectors and these interactions between sectors often work through sectoral objectives. These mechanisms should be considered when designing future HiAP initiatives and their implementation to enhance the emergence of non-health sector policy-makers’ engagement.

## Background

Health in All Policies (HiAP) includes a broad range of approaches aiming to embed systematic action to improve the health impacts of policies beyond the healthcare sector. In the 2011 Rio Declaration, 165 countries committed to implementing HiAP to improve the prevention of disease by addressing the social determinants of health such as housing, education and transportation [1]. The main principles of HiAP are not new, as the 1978 Alma-Ata Declaration already emphasised that “*… the highest possible level of health is a most important world-wide social goal whose realization requires the action of many other social and economic sectors in addition to the health sector*” [2]. However, much of the research about HiAP implementation is descriptive, and there have been calls for more evaluative evidence to explain how and why successes and failures have occurred [3, 4].

A recent study by Molnar et al. [5] examined different strategies for engaging policy-makers from diverse sectors in the implementation of HiAP. The authors found particular support for using win–win strategies (in which both parties gain advantage, as opposed to a win–lose strategy or zero-sum game) to motivate policy-makers across sectors to implement HiAP. Several mechanisms to explain how and why win–win strategies worked were uncovered, including how development of a shared language facilitated communication between sectors, how embedding multiple outcomes into projects helped to appeal to the interests of diverse policy sectors, and how conditions were created to incentivise the adoption of public-health objectives across policy sectors.

Previous studies support these findings. The development and use of shared terminology was essential in implementing the Public Health Act in Norway [6] while, in other cases, intersectoral action benefited from using language about ‘community’ and ‘social sustainability’ instead of ‘health’ [7, 8], and tools focused on human impact assessment and overall policy appraisal instead of health impact assessment [9, 10]. Multiple outcomes have been successfully used in Finland in arguing sectoral objectives such as wealth and economic growth [11] and prime examples of successfully using public-health arguments can be found among road safety policies and tobacco [12, 13]. However, beside Molnar et al. [5], there are no studies that have aimed to examine how and why win–win strategies work across different jurisdictions.

### Systems framework for studying HiAP implementation

In this paper, we test hypotheses about win–win strategies for engaging policy-makers in HiAP implementation drawing on components identified in our systems theory about the implementation of HiAP (Table 1) [14]. Systems theory is often used in policy studies to provide advice about engaging in policy-making [15, 16]. It warns against the assumption of law-like behaviour and the idea that success in one context will mean success in another. The idea of emergence (that a system may have properties that its components do not have on their own) is also particularly significant for HiAP implementation because it highlights macro-social outcomes based on interactions between many actors [17]. Our systems framework helps us focus on specific features of a government, in which individual policy-makers operate together with extra-governmental influences. It presents HiAP implementation as a dynamic set of processes comprising interactions between government subsystems and system components, resulting in the emergence of significant outcomes of HiAP implementation. In total, we use three subsystems (executive, intersectoral, intrasectoral) and eight system components (policy agenda, expert advisors, HiAP management, high-ranking civil servants, sectoral objectives, sectoral ideology, workforce capacity for intersectoral action, workforce HiAP awareness). In practice, we summarise all our empirical evidence as it relates to the systems framework. From a systems perspective, HiAP implementation is about combining different elements of a government system to enhance emergence and engaging non-health sector policy-makers is largely about removing possible barriers for collaboration among different actors [18].

**Table 1:** Eight key system components within three government subsystems (modified from Shankardass et al. 2018 [14])

Subsystem	Component
Executive subsystem: the processes of government responsible for the creation and implementation of legislative mandates related to the implementation of Health in All Policies (HiAP) initiatives	Policy agenda: the finite set of social and political issues upon which governments act on at a given point in time, which will be shaped by the party organisation(s) who control the government and influenced by extra-governmental factors, and which have implications for the priority of health equity initiatives like HiAP
Intersectoral subsystem: the processes of government that facilitate the horizontal and vertical coordination of the HiAP policy agenda across various sectors of the government and with extra-governmental partners	Expert advisors: expert individuals (often from outside of government) who are formally consulted in planning and executing the implementation of HiAP initiatives; expert advisors are a type of policy elite, i.e. they have influence over the policy process HiAP management: the set of technical processes through which governments generate institutional capacity for implementation of HiAP initiatives
Intrasectoral subsystem: the processes of government that facilitate activities such as the pursuit of sectoral objectives, which may be affected by the implementation of HiAP initiatives	High-ranking civil servants: bureaucrats who may have authority over the policy process delegated to them by political elites; high-ranking civil servants are a type of policy elite, i.e. they have influence over the policy process, and may be particularly engaged in the technical aspects of implementing HiAP initiatives Sectoral objectives: goals and motivations of policy sectors, often delivered through a formal mandate from the executive, which may be affected by a government’s implementation of HiAP initiatives Sectoral ideology: the cluster of ideas, beliefs, values and attitudes that constitute the normative lens through which policy-makers within a given sector interpret and act upon social and political issues, such as health equity, and which may vary given sectoral objectives (e.g. healthcare, population health, economic growth, engineering), i.e. a worldview Workforce capacity for Intersectoral Action (ISA): the extent of expertise among human resources with tools and processes and workforce size dedicated to implementing HiAP initiatives, enabling feasibility Workforce HiAP awareness: an understanding of the need and reasons for an intersectoral approach to address health equity, as part of the process of agenda-setting and, ultimately, buy-in for the implementation of HiAP initiatives

We test hypotheses on the mechanisms underlying win–win strategies using our systems framework, together with realist methods. Critical realism, the metatheory behind realist methods, holds that all social phenomena, including policy-making, are explained in underlying causal mechanisms derived from natural and human factors, and because of the myriad relationships among these factors, critical realists view policy-making as complex [19]. Drawing upon realism, we assume that we can uncover mechanisms for engaging policy-makers by studying objective interventions on an empirical sample of six state- and national-level governments (California, Ecuador, Finland, Norway, Scotland and Thailand).

### Hypotheses on using win–win strategies in engaging policy-makers to implement HiAP

For this study, three hypotheses about the mechanisms that explain the win–win strategy were developed based on previous findings from Molnar et al. [5], who identified the salience of the win–win strategy in three cases of HiAP (Sweden, Quebec and South Australia) and described a series of underlying mechanisms that facilitated HiAP implementation. These hypotheses were further honed by consulting with policy-makers working with HiAP, and by reviewing several political and policy science theories [20, 21]. It is important to note that these hypotheses are not mutually exclusive but might occur concurrently in implementing HiAP.

Hypothesis 1: Intersectoral engagement in HiAP implementation requires a win–win approach, reached by understanding the mission and culture of other sectors and developing shared language, because it facilitates buy-in/acceptability for the HiAP strategy.

Hypothesis 2: Intersectoral engagement in HiAP implementation requires a win–win approach, reached by using multiple outcomes to engage non-health sectors because it facilitates buy-in/acceptability for the HiAP strategy.

Hypothesis 3: Intersectoral engagement in HiAP implementation requires a win–win approach, reached by teaching sectors to use public-health arguments to make a stronger case, because it facilitates buy-in/acceptability for the HiAP strategy.

### Predictions about the systems framework

Our hypotheses suggest that all the win–win strategies would facilitate buy-in of different sectors for HiAP implementation. Based on our systems framework, we expected that this would occur through a need by the lead sector (i.e. HiAP management) to (1) understand the mission/culture of participating sectors (i.e. sectoral objectives) and develop a shared language (i.e. sectoral ideology, workforce HiAP awareness), (2) use dual outcomes (i.e. HiAP management and sectoral objectives), and/or (3) use public-health arguments (i.e. HiAP management, sectoral objectives and workforce capacity) to convince sectors to participate.

## Methods

### Explanatory multiple-case study method

A barrier to systematic research on HiAP implementation has been the absence of methods to study complex multisectoral policy interventions [3]. In the HARMONICS project, we used a multiple explanatory case-study approach that deals with complexity by proposing testable hypotheses and telling a “*story of a sequence of events or processes*” that emphasises contextual factors [22]. Our case-study methodology has been described elsewhere [23], but we include a summary of steps for conducting single-case studies and cross-case analysis in Table 2. Essentially, this approach encompasses single, explanatory case studies in which we focus on learning about mechanisms that explain progress (or lack thereof) in the implementation of HiAP, as well as cross-case analysis in which we test our study hypotheses to develop an understanding of how implementation works across case settings. In our analysis, we focus specifically on social mechanisms that involve at least two people with a political, cultural or economic relationship [24] and that are interactive, often hidden processes, which cause HiAP implementation.

**Table 2:** Activities associated with each step of the case study process (modified from Molnar et al. [5])

Activities	Description
Collect and synthesise data within each case and generate single case study reports	
Consult literature	We collected literature on HiAP for each case by undertaking a systematic search for peer-reviewed, government and grey literature that was relevant for the testing of hypotheses
Conduct key informant interviews with Health in All Policies (HiAP) experts	We identified HiAP experts with substantial experience in working on HiAP by undertaking a search for prominent authors of reports on the case as well as by snowball sampling. Expertise and experience were confirmed through screening potential interviewees and, within each case, we interviewed 10–15 individuals representing civil servants from various sectors, politicians and researchers; we inquired about evidence related to the hypotheses using a semi-structured interview guide (the hypotheses as such were not mentioned) and transcribed the interviews for the systematic coding of the data
Code literature and interview transcripts for evidence on hypotheses	We coded and summarised all interview transcripts and literature for evidence of the hypotheses, specifically looking for data on context–mechanism–outcome (CMO) configurations; within each CMO, we indicated whether they directly confirmed or refuted the hypothesis, or whether they served as counterfactual evidence; in particular, we considered how and why certain actions and activities were effective in convincing stakeholders to participate in HiAP initiatives, paying considerable attention to contextual factors that conditioned the mechanisms at play
Summarise findings	Thick interview/literature CMOs (i.e. those with clear links between a mechanism and an outcome) were summarised by a hypothesis; thin interview/literature CMOs (i.e. unclear links between a mechanism and an outcome) were used as supporting evidence
Assess for quality and strength of evidence	We described evidence according to triangulation (i.e. whether the mechanism was supported by both interview and literature sources)
Write case reports	Our end-product for case-specific analyses was a case report
Analyse data across cases	
Synthesise findings for each hypothesis across single case study reports to draw cross-case conclusions	Use results on support for hypothesis from single case studies to (1) categorise cases as literal replications or theoretical replications for each hypothesis and (2) synthesise findings for each hypothesis across cases to draw cross-case conclusions; undertake member checking by sharing findings with advisory group

### Case selection and data collection

Four cases were selected from a scoping review that identified 16 HiAP initiatives implemented globally between 1980 and 2009 [25], followed by an updated literature search in 2014 that further identified two cases. Identified HiAP initiatives included implementation in diverse settings, encompassing jurisdictions in the Asia-Pacific region, Europe, and North and South America. In selecting cases, our purpose was to represent diverse mandates for HiAP implementation. We also aimed to select a combination of high-income environments (Finland, Norway, Scotland, and the US [California]), as well as lower- and middle-income environments (Ecuador and Thailand).

We used two sources of data, namely key informant interviews and peer-reviewed and grey literature. To identify potential key informants, we used purposeful sampling and the snowball method. We began by reviewing extant literature to generate a preliminary understanding of HiAP implementation in each setting. From the literature, we identified both eminent HiAP scholars as well as policy-makers and experts described as essential for each case. In selecting key informants for interviews, we sought a diverse sample in terms of policy sectors and positions, including participants from outside of government as well as referrals from interviewees. All potential informants were first screened for eligibility, and those rating themselves as familiar with HiAP implementation were deemed eligible. For each case, between 10 and 15 interviews were completed, consisting of politicians, civil servants, public-health experts, academics and/or representatives of non-governmental organisations. In all six cases, the interviewed politicians and civil servants represented several different policy sectors (e.g. education, environment, employment, finance).

All potential interviewees received an information sheet by email describing the study, and we obtained their verbal, informed consent prior to the interviews. We created a semi-structured interview guide to collect evidence on our hypotheses (including motivation), with the goal of probing for mechanisms while trying to avoid leading key informants toward any one hypothesis. Phone or face-to-face interviews with key informants were conducted in their native languages and the interviews were audio recorded. A researcher conducted the Finnish interviews while professional interviewers conducted the interviews for the other five cases. The bilingual interviewers (and a Finnish researcher) then transcribed the interviews and translated them to English. The St. Michael’s Hospital Research Ethics Board (#10–162) approved the study and its procedures.

Regarding extant literature, we undertook a systematic search for evidence on our hypotheses by following a predesigned protocol. Grey sources of literature included legislation, reports, guides, website text, tools, meeting agendas, presentations, recommendations and/or briefing notes from various government and academic institutions.

### Analysis

Using a protocol [23, 26], we created what Pawson and Tilley [27] call context–mechanism–outcome (CMO) pattern configurations to articulate mechanisms that explain how complex programmes work and fail in different contexts. CMOs are the cornerstone of Pawson and Tilley’s realistic evaluation and focus on the relationship between the context of participants’ lives (what conditions are needed to trigger a mechanism to produce an outcome), the underlying causal mechanisms (how a mechanism leads to a particular outcome in a given context) and the outcomes (what outcomes are produced by mechanisms triggered in a given context).

For each of our six cases, two investigators independently coded interview data (using comments in Microsoft Word) to flag text that directly referred to our hypotheses, focusing on rich descriptions of mechanisms. In teams of two or more, we reviewed all coded interview data to generate CMO-pattern configurations and indicate whether they confirmed or refuted our hypotheses. Due to human-resource and time constraints, literature data were coded and summarised by one person (using comments in Adobe Acrobat). This was not a major impediment to analysis, as extant literature on mechanisms was often sparse. All data were stored electronically in an Excel database. Files were stored in clearly organised and labelled folders and filed in a shared drive to which only the research team had access.

For each case, using the CMO database, we summarised all thick evidence (i.e. the CMOs with clear links between a mechanism and an outcome) as it related to particular hypotheses (see Appendix as an example of a narrative evidence summary for one case). During this stage, we paid particular attention to the relationship between our evidence and the systems framework. In preparation for the cross-case analyses, we assessed which system component we expected to be relevant for a given hypothesis. We then applied our evidence for all cases to the systems framework to assess the extent to which our predictions about the systems framework were represented in the evidence. In the results section, the system components are indicated using quotation marks.

We assessed the quality of evidence within and across cases in terms of triangulation of sources and strength of evidence (Table 3). We also strengthened hypothesis testing using replication logic. Replication logic means that, because of anticipatable reasons (i.e. case characteristics), each case predicts either similar (a literal replication) or contrasting (a theoretical replication) results [26]. Using information on HiAP mandates in different jurisdictions, we identified an index case (Ecuador) expected to provide strong support and then classified every comparison case study as either a literal or theoretical replication. Using literal replications, we tested situations in which causal factors on the implementation of HiAP were expected to produce the same results, and we used theoretical replications when contrasting results were expected, based on differences in the HiAP mandates (Table 4). As we considered engaging non-health sector policy-makers by using win–win strategies to be largely about removing possible barriers for collaboration among different actors, we defined the cases with strong commitment to a HiAP mandate as literal replications (e.g. intersectoral council and specific funding allocated to the implementation) and the cases with weak commitment to the mandate as theoretical replications. Our replication logic also holds that the clarity of mandate (e.g. clear targets for monitoring the implementation) would not matter in using win–win strategies.

**Table 3:** Quality of evidence within and across cases

*Triangulation*	*Evidence that is supported by multiple sources (i.e. literature and interviews); assessed at single case stage of analysis*^*a*^
Strong	Thick evidence from three or more sources of data (e.g. literature or different types of informants)
Adequate	Thick evidence from two sources of data (e.g. literature or different types of informants)
Limited	Thick evidence from only one source of data (i.e. literature or type of informant)
Thin evidence only	Only thin evidence available
No evidence	No evidence was generated
*Strength of Evidence Across Cases*	*The degree of support for the hypotheses within either the literal or contrast replication for a given hypothesis across cases; assessed at cross-case stage of analysis*
High	Support is high when triangulation is at least adequate across 60% or more of cases
Medium	Support is medium when triangulation is at least adequate across 40% of cases
Low	Support is low when there is less than 40% adequacy
Thin evidence only	Only thin evidence available
No support	No thick or thin evidence was found (i.e. the hypothesis was not discussed by key informants or in the literature)

^a^ Different types of key informants (e.g. civil servants in different sectors, such as health and transportation, politicians, activists/advocates, academics) can be considered a unique type of data/‘source’

**Table 4:** Replication Logic

Indicator	Strength of commitment to the Health in All Policies (HiAP) mandate	Clarity/detail of mandate
California (Theoretical replication)	Weak Mandate is an Executive Order with mechanisms in place to ensure accountability; a HiAP Task Force was established to promote the mandate No public funds available for specific initiatives	Unclear Targets and timelines on ad hoc basis benefiting mutual partners through the identification of strategies that address multiple goals at one time while providing ‘co-benefits’; the Department of Health is responsible for implementation, including setting priorities and facilitating ISA; the goal of improving population health through promoting equity and sustainability
Ecuador (Literal replication)	Strong Mandate is a long-term strategy called ‘Buen Vivir’, backed by the 2008 Constitution with specifically provided targets and timelines; the National Secretariat for Planning and Development (SENPLADES) is the entity that promotes the country’s integrated development at the national and sector-wide levels; funds for initiatives come from the central government	Clear Total of 92 national policies and 138 goals and indicators; HiAP coordinated by SENPLADES at the national level; the National Planning Council (an intersectoral, professional body) serves as the technical secretariat for all levels of government; health is described as an important sector with a work plan (others include green economy, trade, and technology, working together as an overreaching national strategy), helping meet the requirements of “Good Living Objective 3: To Improve the Quality of Life of the Population”
Finland (Theoretical replication)	Weak Mandate is a long-term strategy called ‘Health 2015’; accountability mechanisms are coordinated by the Advisory Board for Public Health and the Ministry of Social Affairs and Health Limited public funds were allocated for initiatives	Clear Health 2015 outlines 8 goals with a 15-year timeline; the Advisory Board for Public Health and the Ministry of Social Affairs and Health coordinate the implementation and monitoring; HiAP’s aim of enabling people to live longer and healthier lives while reducing health inequalities within the country
Norway (Literal replication)	Strong Mandate is a long-term strategy called National Strategy to Reduce Social Inequalities in Health; accountability mechanisms include annual review and reporting; an interministerial committee was created and initiatives were incorporated into the national budget	Clear Objectives clearly stated but without clear timelines; the Directorate of Health is responsible for coordinating sectors and monitoring/reporting on progress; HiAP is framed around social equity but focused on reducing health inequities
Scotland (Theoretical replication)	Weak Mandate were pilot strategies called ‘Equally Well’ and ‘Achieving our Potential’; accountability mechanisms include reporting and evaluation of Equally Well conducted by local test sites and the Ministerial Task Force; a Ministerial Task Force on Health Inequalities was created to support HiAP; limited public grant funding for pilot projects was provided	Clear Achieving our Potential includes specific targets; Equally Well does not provide clear targets and timelines; regular evaluations at a set timeline were laid out, without targets (although the nature of test site work including emergent objectives/activities renders that somewhat irrelevant); each Equally Well test site had a coordinator Ministerial Task Force responsible for evaluation; Equally Well is clear about improving health equity
Thailand (Literal replication)	Strong Mandate derives from the National Health Act; accountability mechanisms derived from the Thai constitution include the public’s right to sue government organisations that fail to comply with regulations about impact assessment; a National Health Assembly and National Health Commission were created to support HiAP	Unclear Health Impact Assessment (HIA) is a key part of policy coordination in Thailand (however, no clear targets or timelines for the use of HIA were found); the National Health Commission, which is an intersectoral governmental body, approves a budget specific for the National Health Commission Office, which is largely responsible for HiAP-related activities in Thailand; one principle of HIA use in the National Health Act is justice in order to “*reduce inequity and injustice in respect of health*”

## Results

### Shared-language win–win strategy

As shown in Table 5, across the six cases, there was a high degree of support indicating that the shared-language win–win strategy facilitated sectoral buy-in, with no evidence against it. We found strong support in California and Scotland, while support was adequate in Ecuador and Norway, but limited for Finland and Thailand. All of the strong evidence was found for theoretical replications (California and Scotland), while all adequate evidence was found for literal replications (Ecuador and Norway).

**Table 5:** Evidence on three win–win strategies from six governments

	For		Against	
Shared language win–win strategy				
Case	Within	Across	Within	Across
California	Strong	High	No evidence	No support
Ecuador	Adequate		No evidence	
Finland	Limited		No evidence	
Norway	Adequate		No evidence	
Scotland	Strong		No evidence	
Thailand	Limited		No evidence	
Multiple outcomes win–win strategy				
Case	Within	Across	Within	Across
California	Strong	High	No evidence	No support
Ecuador	Limited		No evidence	
Finland	Adequate		No evidence	
Norway	Strong		No evidence	
Scotland	Strong		No evidence	
Thailand	Adequate		No evidence	
Public health arguments win–win strategy				
Case	Within	Across	Within	Across
California	Limited	Low	No evidence	No support
Ecuador	No evidence		No evidence	
Finland	Strong		Thin evidence	
Norway	No evidence		No evidence	
Scotland	No evidence		No evidence	
Thailand	No evidence		No evidence	

For example, in Scotland, the shared-language win–win strategy was effective at garnering buy-in for planning HiAP interventions at the national and local levels by facilitating a mutual understanding (‘workforce capacity for ISA’) of different ‘sectoral objectives’ and creating a supportive, more trusting environment for developing planning partnerships. In practice, the Equally Well strategy as such already focused on well-being rather than health and, at an early phase of the implementation, learning sites were created. According to one informant, the learning sites made possible that “*people could talk about the different approaches and learn the different languages*” across sectors, such as the built environment and health.

### Multiple outcomes win–win strategy

A high degree of support was also found for the multiple outcomes win–win strategy facilitating buy-in, with no evidence found against it. California and Scotland (theoretical) as well as Norway (literal) provided strong support for our hypothesis, whereas Finland (theoretical) and Thailand (literal) provided adequate evidence, and Ecuador (literal) provided limited evidence.

For example, in California, an ‘expert advisors’ consultative approach was used successfully to identify collaborations based on existing objectives, giving the sectors a feeling that they were addressing their own goals (‘sectoral objectives’) distinct from health, such as liveability and sustainability. Specifically in sectors where there was initial resistance, this approach was beneficial in showing the merits of structured intersectoral action on achieving each sectors’ own objectives.

### Public health arguments win–win strategy

For the public health arguments win–win strategy, the degree of support across cases was low, although there was no support against it either. We only found strong evidence supporting our hypothesis from Finland, and limited evidence from California, both representing theoretical replications. In Finland, austerity (‘policy agenda’) reduced support for environmental protection as the government claimed that they could not afford to favour environment over employment and economic growth. In this situation, the Ministry of the Environment (‘high-ranking civil servants’) collaborated on health objectives (‘sectoral objectives’) to get more people to buy into the environmental protection agenda, that at times conflicts with the objectives of the industry.

### Predictions about systems framework

In relation to our a priori expectations about how our systems framework could help indicate mechanisms related to the win–win strategies, we found sectoral objectives to be the only component relevant across all cases. In Scotland, Thailand and Finland, non-health sectors (e.g. mining and environment) realised the benefits of addressing health and health inequalities to achieving their own sectoral objectives, which facilitated buy-in for HiAP implementation. In Norway, the education sector decided to prioritise health (e.g. to invest in ventilation systems and mental health promotion in schools) when recognising its importance for their own sectoral objectives (i.e. student learning outcomes) and engaging non-health sectors on implementing the National Strategy to Reduce Social Inequalities in Health improved after the health sector invited other sectors to incorporate their objectives into the process. In California, sectoral objectives were used to employ a focus on HiAP activities that benefit the health and non-health sectors alike after expert advisors had identified potential collaborations based on existing sectoral objectives, as described earlier. In Ecuador, the two-tiered governance structure enabled the coordinating ministry (upper tier) to facilitate buy-in for HiAP by using its role laid out in the mandate to help shape the sectoral objectives of lower tiered ministries.

We found little or no support that some systems components were relevant to the underlying mechanisms of the win–win strategy, including sectoral ideology, capacity, awareness and management. In fact, Scotland was the only case in which workforce capacity was relevant, and only one of two — the other being Ecuador — in which HiAP management was observed. Norway and Finland were the only two cases in which awareness proved relevant.

## Discussion

In this cross-case study of six state- and national-level governments, we found robust evidence for two mechanisms about how and why win–win strategies build partnerships for HiAP implementation, namely the use of shared language and the value of multiple outcomes. Within our cases, the triangulation was strong, both hypotheses were supported by literal and theoretical replications, and there was no support against them.

The cornerstone of the shared-language win–win approach is to modify the health-sector terminology and pro-HiAP arguments to engage with the language of the audience. Our evidence for the effectiveness of this strategy in different jurisdictions seems understandable when we consider that (1) policy-makers often interpret key public-policy terms in different ways [28], (2) within different government systems and sub-systems, policy-makers also use some of their own unique languages [17], and (3) the use of specific terminology (e.g. health and equity) includes beliefs and values that have the potential to conflict with the understanding of policy-makers representing other sectors [28]. Furthermore, all of the strong evidence for using the shared-language win–win approach was found for contrast replications, indicating that strong commitment for the HiAP mandate is not a requisite for this strategy to be effective (neither does it matter whether the mandate is clear or unclear).

The dual-outcomes win–win strategy is based on an understanding that governments contain systems and subsystems with their own rules on what to pursue and how to pursue it [29] and that, as power is shared across many government sectors and levels of government, with a range of quasi-governmental and non-governmental actors, policy-makers are, to a varying degree, dependent on other actors who exert some degree of power over policy outcomes [30, 31]. Based on our evidence, recognising how one is situated within a government system, and utilising this information in negotiating actions and dual outcomes, works in engaging policy-makers in different settings.

In using the public health arguments win–win strategy, we only found evidence from Finland, where non-health sectors bought into HiAP to advance their own sectoral objectives. Specifically, the Ministry of Environment used public-health arguments to “*frame*” [32, 33] their own objectives and actions. Using public health arguments, the Ministry of Environment gained support from the Ministry of Social Affairs and Health (which they considered to be more powerful) and made their own objectives and actions easier to engage with for a wide range of policy-makers throughout the government system. The fact that we only found evidence on using public health arguments to advance non-health sectors’ own objectives from Finland might be due partly to the historical legacy and institutionalisation of intersectoral action in the Finnish context [34–36]. This legacy could also help to explain why there was relatively little evidence that buy-in for HiAP required the development of a shared language, since non-health sectors may already understand the rationale for HiAP and their role in the approach.

In their study, Molnar et al. [5] found stronger support for the use of the public health arguments win–win strategy. Compared with their study, whose sample included Quebec, South Australia and Sweden, our study captured a larger sample of cases from relatively different contexts (high-income and middle-income settings, different types of mandates). In both studies, the collection and analysis of data were based on using a well-designed protocol [23, 26] that enabled the gathering of detailed explanations of implementation outcomes. In both studies, key informants were also selected carefully from within and outside of government, and they were guaranteed full anonymity, increasing the opportunity to learn about HiAP implementation from diverse perspectives and without a filter.

However, it should be noted that, for either study, it was not possible to recruit key informants from all policy sectors involved and from all geographical levels, for all cases. Both studies might also face limitations because of key informants’ limited experiences with facilitators and barriers of HiAP implementation. After all, in both studies, there were only 10–15 key informants for each case, the literature was often sparse concerning mechanisms, and either study had no explicit way of assessing the importance of different outcomes objectively (e.g. small vs. large). These methodological limitations might partly explain the varying results between studies regarding the public health arguments win–win strategy, together with contextual factors producing different results for different samples of cases.

Obtaining comparable qualitative information might also have suffered from cultural differences and the fact that our six cases represent five different native languages. However, we have no means to assess the scope or strength of this effect. Even if our results are not generalisable to other contexts, they reveal the inner workings of win–win mechanisms in six different contexts and provide important insights into designing future HiAP initiatives and their implementation.

We have previously argued that a systems framework would be useful for understanding HiAP implementation [14], and others have espoused systems theory to explain engagement in policy-making [15, 16]. Based on our systems framework, we expected that the most important system components to using win–win strategies are sectoral objectives, and we found empirical support for this prediction. Our theoretical insight here was that policy-makers’ engagement with HiAP implementation is strongly influenced by how they situate into a government system, and that different government sectors include their own objectives, each providing different incentives for policy-makers to engage with particular actions. However, we also expected that sectoral ideology would play a considerable role but found very little support for this. This is somewhat surprising, as previous studies have indicated that policy-makers create coalitions with actors, and they are influenced the most by actors who understand their ideologies, despite formal organisational arrangements and boundaries within a government system [30, 37]. In future studies, it is worth explicitly addressing specific ideological assumptions to test how sectoral ideologies vary and if they really do not play a role in using win–win strategies in engaging policy-makers to implement HiAP [38].

## Conclusions

We conclude that two mechanisms about how and why win–win strategies build partnerships for HiAP implementation, namely the use of shared language and the value of multiple outcomes, were found as relevant to six settings (California, Ecuador, Finland, Norway, Scotland and Thailand). Both of these mechanisms trigger a process of developing synergies and releasing potentialities among different government sectors and these interactions between sectors often work through sectoral objectives. These mechanisms should be considered in designing future HiAP initiatives and their implementation to enhance the emergence of non-health sector policy-makers’ engagement. For the use of the public health arguments mechanism, we only found evidence from Finland, and conclude that this might be due partly to the historical legacy and institutionalisation of intersectoral action in the Finnish context.

## Data Availability

The datasets generated and/or the summary tables from our analysis during the current study are not publicly available due to the sensitivity of confidential interviews with government employees, but they are available from the corresponding author in a de-identified manner upon reasonable request. The semi-structured interview guide is also available from the corresponding author.

## Appendix

### Narrative evidence summary for Norway

#### Strong evidence for multiple outcomes win–win strategy

Strong evidence described the importance of using a win–win multiple outcomes approach in engaging non-health sectors. One thick mechanism described how ‘de-healthifying’ language helped other sectors to see Health in All Policies (HiAP) as an advantage to support their own objectives, engaging non-health sectors at the national level (outcome: non-health sector buy-in). Another indicated that the school sector decided to prioritise health (outcome: non-health sector buy-in) on a local level when recognising the importance of health for learning as well as the importance of the school environment for health. The informant stated “*If you’re in the schooling system, your main priority is to get school instructions or whatever, but they are very aware of mental health because without good mental health there is no learning*” (NOR177_04). Finally, one informant described how engaging non-health sectors on implementing the National Strategy to Reduce Social Inequalities in Health on a national level improved after the health sector invited other sectors to incorporate their own goals and expertise into the process.

#### Adequate evidence for shared-language win–win strategy

Adequate evidence described the importance of understanding the mission and culture of non-health sectors and using a shared-language win–win approach. One thick CMO indicated that buy-in (Outcome) of non-health sectors improved on a national level after becoming aware of the need to work together to obtain each sector’s missions and sectoral objectives. We also found one evidence describing how buy-in of non-health sectors improved on a national level after the development of a common understanding of HiAP goals. One interview indicated how creating an understanding of health inequalities by describing health in terms of quality of life (and avoiding the WHO definition of health) helped to engage municipalities. “*Common understanding around what is common language to discuss things because talking about health I think many people have different perspectives on what is health and we now try to develop a common set of understandings*” (NOR117_06).

## Abbreviations

CMO: context–mechanism–outcome
HARMONICS: HiAP Analysis using Realist Methods On International Case Studies
HiAP: Health in All Policies
